# Post-ICP Chemical Ionization MS for Total Extractable Organic Fluorine Quantitation

**DOI:** 10.1021/acsomega.4c09483

**Published:** 2024-11-05

**Authors:** Samuel White, Kaveh Jorabchi

**Affiliations:** Department of Chemistry, Georgetown University, Washington, District of Columbia 20057, United States

## Abstract

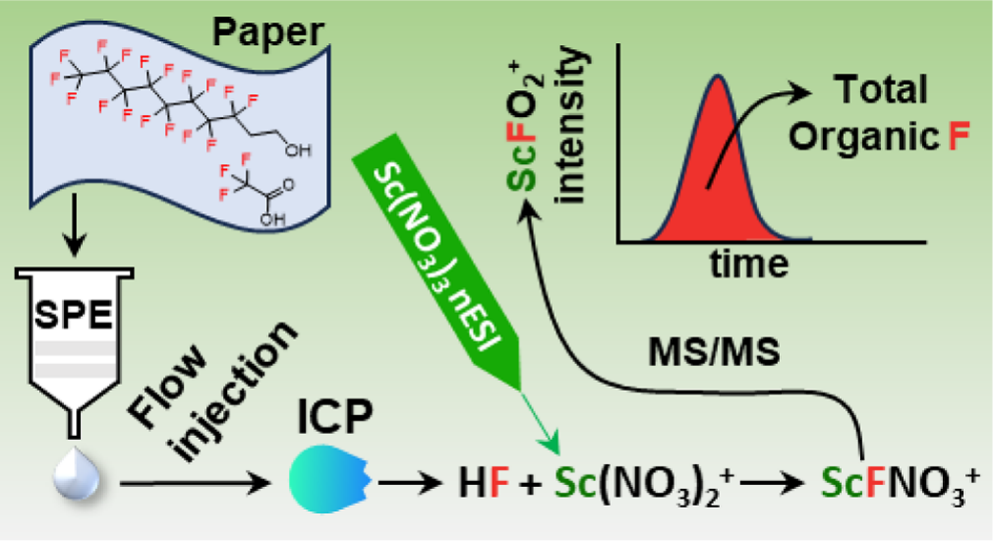

Total extractable organic fluorine (EOF) quantitation complements targeted analyses to ensure complete accounting of all fluorochemicals in a sample. Notably, the prevalence of perfluoroalkyl substances has increased the need for total EOF quantitation due to the myriad forms of these chemicals and the limited availability of standards for targeted analysis. Here, we evaluate postplasma chemical ionization mass spectrometry (MS), an approach developed to address the limitations of inductively coupled plasma (ICP)-MS in F analysis, for total EOF quantitation. Organofluorine-containing samples are introduced into an ICP, leading to the post-ICP formation of HF, which is then ionized to ScFNO_3_(H_2_O)_*n*_^+^ and detected by quadrupole MS. We report optimal F detection across a wide range of ICP operating parameters, highlighting robust ion generation processes. We then compare sample introduction methods and show that a single-pass spray chamber mitigates volatility-related biases, yielding compound-independent F response factors and an instrumental detection limit of 2.2 ng of F mL^–1^ by using flow injections. This detection limit is 10-fold better than that offered by ICP-MS and comparable to that of combustion ion chromatography. Subsequently, we conduct total EOF quantitation of food contact paper products via methanolic extraction of 3 cm × 3 cm samples followed by 8-fold preconcentration with solid phase extraction and flow injections, achieving a detection limit of 1.2 ng F cm^–2^. We show that organic fluorine is detected on all 9 locally collected food contact paper products with total EOF contents of 22–363 ng F cm^–2^. These studies expand elemental MS capabilities in F analysis and highlight post-ICP chemical ionization MS as a facile total EOF quantitation technique.

## 1 Introduction

The carbon–fluorine bond in per- and polyfluoroalkyl substances (PFAS) provides advantageous chemical and physical properties, including high stability, hydrophobicity, and lipophobicity, granting PFAS widespread utility.^1^ However, associations have been established between PFAS exposure and health risks, such as cancer, pregnancy-related issues (e.g., low birth weight and hypertension), and developmental issues in children.^2−4^ These concerns along with the growing number of PFAS (>14,000 chemicals cataloged by the US Environmental Protection Agency)^5^ necessitate the development of detection and quantitation methods adaptable to the evolving analytical needs.

Currently, PFAS quantitation is conducted in two complementary modes: (1) targeted quantitation of analytes and (2) total F quantitation. The targeted methods provide good performance via PFAS extraction from matrix followed by chromatography coupled to MS.^6−8^ However, only a small portion of the total PFAS (typically up to 40 PFAS compounds) is monitored in these methods due to a lack of standards. On the other hand, the total F atom concentration in the extract can be determined by elemental techniques without compound-specific standards. This is termed total extractable organic fluorine (EOF) and offers a mass balance check for targeted quantitation. Notably, several studies have indicated that the sum of PFAS quantified in the targeted methods accounts for a small fraction (∼10%) of the total EOF, highlighting the limitations of the targeted methods and the significance of total EOF measurements.^6−9^ Further, total EOF quantitation may serve as a rapid screening technique to identify samples with high contamination load, and to monitor byproduct generation in PFAS destruction technologies.^6,10^ Relatedly, new toxicity thresholds are proposed based on total organic F as opposed to a threshold for each compound. For example, the Danish Ministry of the Environment and Food places an indicator value on food contact paper products with fluorine content exceeding 100 ng organic F cm^–2^ (or 20 μg organic F per g of the paper).^11^ With these applications, the demand for total EOF quantitation techniques has increased in recent years.

In response to this increasing demand, various elemental analysis techniques have been tested for total EOF quantitation, each presenting their specific advantages and challenges, as noted below. Particle-induced γ-ray emission spectroscopy (PIGE) is a surface analysis technique adaptable for EOF quantitation via collecting PFAS on a sorbent.^12^ Instrumental limits of detection (LOD) of 250–300 ng F cm^–2^ have been reported for PIGE,^13,14^ corresponding to absolute LOD > 100 ng F in the analyzed sample.^9,12^ This mandates a relatively large sample size. Such sample sizes are available for water analysis,^12^ however, biological samples may be limited in total absolute F amount, e.g., blood with extractable fluorine concentrations <20 ng F mL^–1^,^15,16^ leading to challenges in total F quantitation using PIGE.^19^ F-NMR is another spectroscopic technique that was recently proposed as an approximate method for total PFAS quantitation in solutions. This method takes advantage of the constant F chemical shift of the terminal CF_3_ in majority of PFAS with carbon chain lengths >6.^17,18^ An instrumental LOD of 100 nM terminal CF_3_ is reported using perfluorooctanesulfonic acid, corresponding to 32.5 ng F mL^–1^ (absolute LOD ∼ 16 ng F with 0.5 mL solution in NMR tube). In addition to the need for large sample size, the method has lengthy analysis time (>7 h per sample to achieve the above LOD),^17^ severely limiting throughput.

Combustion ion chromatography (CIC) is currently the most often used technique for total EOF analysis owing to relatively fast analysis and good instrumental LODs as low as 3 ng F mL^–1^.^16,19^ Combusted sample amounts >200 μL are often used, resulting in absolute LODs > 0.6 ng F, better than the methods noted above. However, combustion efficiencies may vary between compounds, causing some uncertainties in accuracy of total F quantitation.^7,20^ Graphite furnace high-resolution molecular absorption spectroscopy (GF-HR-MAS) is a related technique where F atoms are converted to gaseous molecular species (e.g., GaF) and detected by absorption spectroscopy. Instrumental LODs of 0.43–0.8 ng F mL^–1^, corresponding to absolute F amounts of 0.007–0.013 ng,^19,21^ have been reported with this technique, indicating a better performance compared to CIC. However, compound-dependent response arising from complex thermal degradation reactions on furnace surface has been reported for GF-HR-MAS.^22,23^ Compound volatility also significantly affects the F response in GF-HR-MAS,^23^ limiting the accuracy of methods based on this technique for broad total organic F quantitation. Combustion coupled to fluoride ion selective electrode has also been examined for total organic F analysis, albeit with inferior instrumental detection limits (100 ng F mL^–1^) compared to techniques above.^24^ The species-independent response in this method is also yet to be examined.

A common drawback of the aforementioned F analysis techniques is their batch-mode operation. In contrast, continuous-flow operation would provide not only facile total F analysis via flow injections but also quantitation of each analyte without a compound-specific standard via online coupling to liquid chromatography. Notably, plasma-based MS techniques are promising in this regard, with inductively coupled plasma (ICP)-MS as the most commonly used approach. Conventionally, singly charged positive atomic ions are created in ICP-MS for elemental detection, however, poor F^+^ formation efficiency (9 × 10^–4^ % at 7500 K)^25^ resulting from fluorine’s high ionization potential (17.42 eV) has required development of alternative ionization chemistries. In one approach, barium is introduced concurrently with fluorochemicals into the ICP to form BaF^+^ inside the plasma, offering instrumental LODs of 22–60 ng F mL^–1^ via continuous flow infusion.^26−28^ However, these LODs are still >1 order of magnitude inferior to that of CIC for total EOF quantitation. Online coupling with LC has also been demonstrated using in-ICP BaF^+^ formation,^29^ though robust compound-independent quantitation is yet to be established.

To overcome the shortcomings of in-plasma ionization for F analysis, we have developed an alternative approach where ICP serves as a chemical reactor to generate HF from organofluorines followed by post-ICP ionization of HF using reagent ions supplied from a nano-ESI. Our previous work on BaF^+^ formation using this approach demonstrated 2 orders of magnitude increased ion detection efficiency relative to that of in-plasma ionization, and offered instrumental LOD of 11 ng F mL^–1^ (corresponding to 0.22 ng F absolute LOD with 20 μL flow injections) using a quadrupole MS.^30^ Recent works using a high-resolution MS further improved the instrumental LOD to 1.8 ng F mL^–1^ (0.09 ng absolute F) while quantitation without compound-specific standards was also achieved when coupled to LC.^31,32^

Importantly, the post-ICP chemical ionization reactions can be tuned for better performance, while such capability is limited in ICP-MS because of the high-temperature in the plasma. Taking advantage of this capability, we have recently reported ScFNO_3_^+^ formation via post-ICP reactions, demonstrating advantages of this ionization chemistry for F detection in the presence of interferences, such as Cl, thus enhancing the robustness of quantitation.^33^ Here, we apply Sc-based ionization to develop a rapid method for total EOF quantitation using flow injections. Notably, a quadrupole-based MS instrument is utilized in this work, improving adaptability of the method due to wide availability of these instruments. We report improved F detection in high organic solvent content suitable for preconcentration of organofluorines. This is achieved via optimization of critical ICP operating parameters, namely, power and aerosol flow rate, as well as sample introduction systems. Compared to our previous report using a quadrupole instrument, a significant improvement in LOD is attained, and a better species-independent response is established to minimize volatility biases. We then utilized solid phase extraction for total EOF quantitation in food contact paper, demonstrating good performance relative to other methods. To the best of our knowledge, this is the first ICP-based total EOF method rivaling that of CIC in LOD while offering facile and rapid flow injection analysis. This method is also complementary to the species-independent quantitation of fluorinated compounds reported in our previous work,^32^ denoting ICP-chemical ionization MS as a noteworthy technique for a wide range of analysis modes in fluorochemical characterization.

## 2 Materials and Methods

### 2.1 ICP-Chemical Ionization-MS

Key experimental parameters are listed in Table S1. Analyte solutions in 80:15:5 ACN:W:concentrated ammonium hydroxide solvent were injected via 30-μL flow injections at 50 μL min^–1^ and nebulized into a spray chamber. The aerosols were then mixed with makeup gas containing argon and oxygen. Two spray chambers, one cyclonic and one single-pass, were used in this work (Figure S1). For the cyclonic spray chamber, the makeup gas was added to aerosol flow by using a tangential tee (Meinhard, CO) downstream of the spray chamber. For the single-pass spray chamber, makeup gas was directly introduced into the spray chamber via the PEEK sheath flow adapter. Oxygen was added to the makeup gas at a constant flow rate of 0.14 L min^–1^ to avoid soot formation and carbon build up on instrument surfaces upon introduction of organic solvents to the ICP. The argon gas flow rate in the makeup gas was varied to optimize the analytical signal intensity. The aerosol flow exiting the spray chambers was directed into an ICP via a 2 mm injector.

**Figure 1:**
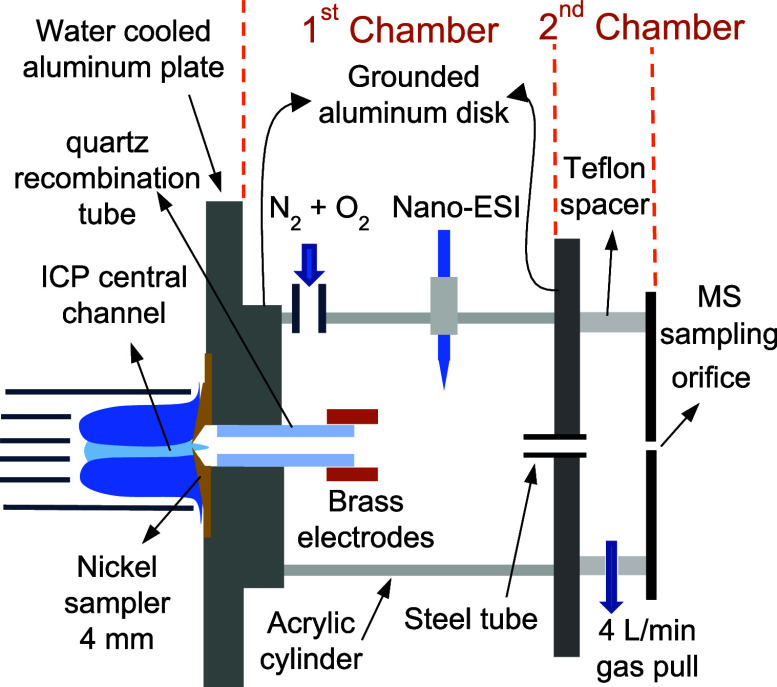
Schematic of post-ICP chemical ionization interface reproduced with modifications from ref.^33^ with permission from the Royal Society of Chemistry.

A two-chamber post-ICP chemical ionization interface schematically depicted in Figure 1 was used in this study, similar to that reported previously.^33^ The central channel of the ICP was sampled by a 4 mm water-cooled nickel cone into the first chamber of the chemical ionization interface. The plasma sampling extent was controlled by two flow rates: (1) a gas evacuation rate of 4 L min^–1^ applied to the second chamber at the downstream end of the interface and (2) 2.56 L min^–1^ of input gas composed of 0.56 L min^–1^ oxygen and 2 L min^–1^ N_2_ supplied to the first chamber at the upstream end of the interface. A higher gas flow rate evacuated from the interface compared to that directed into the interface resulted in sampling the central channel of the plasma through the nickel sampling cone. The plasma flow then passed through a quartz tube within the first chamber, enabling cooling and recombination reactions to form HF from the plasma-degradation products of fluorinated compounds. At the quartz tube’s terminus, two parallel brass plates, one at 400 V and the other grounded, filtered remaining plasma-generated ions and electrons, permitting gaseous HF to proceed toward the MS. The HF was then ionized by reagent ions produced by 1400 V applied to 1 mM scandium nitrate solution in a pulled borosilicate glass nano-ESI capillary. The resultant ions were drawn through a 2 mm i.d. steel tube connecting the first and the second chamber. The second chamber was sealed to the ion sampling plate of the MS. A fraction of the flow carrying ions through the 2 mm steel tube was sampled by the MS while the remaining portion was exhausted into a hood through the port that provided the interface evacuation at 4 L min^–1^.

The nano-ESI produces reagent ions in the form of Sc(NO_3_)_2_(H_2_O)_*n*_^+^, which yield ScFNO_3_(H_2_O)_*n*_^+^ upon reaction with HF in the chemical ionization interface.^33^ While oxygen flow added to the plasma could negatively affect some post-ICP ionization reactions via production of HNO_3_,^32^ the Sc-based ionization is not affected by the presence of HNO_3_, enabling operation at highly oxidative plasma conditions with minimal sensitivity to overabundance of oxygen in the plasma.^33^ Our screening experiments in this work also reflected these earlier findings; therefore, a constant and relatively high (0.14 L min^–1^) flow rate was selected for oxygen, maintaining oxidative conditions with organic solvent introduction. To improve LODs, the ions were monitored using multiple-reaction monitoring. Specifically, ScNO_3_F(H_2_O)_4_^+^ (*m*/*z* 198) was fragmented to ScO_2_F^+^ (*m*/*z* 96) while the reagent ion Sc(NO_3_)_2_(H_2_O)_3_^+^ (*m*/*z* 223) was fragmented to ScNO_3_O_2_^+^ (*m*/*z* 139). The continuous monitoring of the reagent ion was utilized to ensure the stability of the ionization reactions throughout the experiments.

### 2.2 Chemicals

F-containing compounds with wide ranging structures (including PFAS and non-PFAS) were utilized to establish the uniformity of the instrument response for F detection. Fluconazole (FCZ), fludioxonil (FDX), perfluorooctanoic acid (PFOA), perfluorooctanesulfonic acid (PFOS), 1*H*,1*H*-perfluorooctyl methacrylate (PFMC), trifluoroacetic acid (TFA), methyl perfluorooctanoate (MePFOA), floxuridine (FOX), and haloperidol (HAL) were purchased from Sigma-Aldrich (Milwaukee, WI). 1*H*,1*H*,2*H*,2*H*-perfluoro-1-decanol (8:2 FTOH) and decafluorobiphenyl (DFP) were acquired from Oakwood Chemicals (West Columbia, SC). Perfluorobutanoic acid (PFBA) was obtained as 1 mg mL^–1^ standard in methanol from AccuStandards (New Haven, CT). The stock solutions were prepared by dissolving the neat standards in 80:20 acetonitrile (ACN, Fisher Scientific, Fairlawn, NJ):18.2 MΩ cm^–1^ water (W, Elga Purelab Flex, Lane End, UK). Working solutions were prepared by diluting the stock solutions with a final solvent composition of 80:15:5 ACN:W:concentrated ammonium hydroxide (Sigma-Aldrich). Given the interest in total elemental F concentration, the standards were prepared to have a known total F concentration in ng mL^–1^ regardless of the chemical structure and molecular weight of the compounds used for the preparation of the standards.

### 2.3 Organofluorine Extraction from Food Contact Paper

Analyte extraction and sample preparation were based on the procedure reported by Gebbink et al. with modifications.^34^ Nine food contact paper products were collected from establishments around College Park, MD. A 3 cm × 3 cm cutout from each product was placed in 40 mL of methanol (Fisher Scientific Fairlawn, NJ) and lightly shaken for 8 h. To eliminate inorganic F and preconcentrate the sample, a solid phase extraction (SPE) procedure was followed, as described below. This procedure also eliminated easily ionizable elements (e.g., sodium and potassium) from the sample, which can interfere with F detection in plasma-based methods.^27^

To maximize analyte recovery in SPE, we utilized Strata-X-AW (5 mL, 250 mg, 33 μm polymeric weak anion, Phenomenex) and Strata-X (5 mL, 250 mg, 33 μm polymeric reversed phase) cartridges in series with the effluent of the Strata-X cartridge directed into the Strata-X-AW cartridge, providing both hydrophobic and ion exchange mechanisms for analyte retention. Prior to analyte loading, the tandem cartridges were conditioned with 4 mL methanol and then 4 mL acetonitrile, followed by 4 mL of water at a flow rate of 1 mL min^–1^. To increase the retention of the analytes by SPE, the 40 mL of methanolic extract was first diluted with water to 800 mL, reducing the organic solvent fraction. The 800 mL analyte solution was then loaded onto the tandem SPEs at a rate of 1 mL min^–1^ using Phenomenex SPE tube vacuum manifold. The cartridges were then washed with 4 mL of 25 mM ammonium acetate in water at a flow rate of 1 mL min^–1^. The SPE cartridges were subsequently separated and allowed to ambient dry for at least 2 h. After drying, the SPE cartridges were reassembled in tandem and were manually eluted with 4 mL of 80:15:5 ACN:W:concentrated ammonium hydroxide into a 5 mL volumetric flask. The solution was then diluted to 5 mL with more elution solvent. This procedure provided 8-fold preconcentration factor by transferring the analytes from the original 40 mL methanolic extract to a final volume of 5 mL in SPE elution solvent.

## 3 Results and Discussion

### 3.1 Effect of Plasma Operating Parameters on Analytical Ion Signal

To optimize the ion detection sensitivity, we first investigated the effects of two critical plasma operating parameters, namely, the total aerosol gas flow rate and ICP RF power. These parameters affect plasma properties, which may also alter the plasma-induced degradation efficiency of compounds and uniformity of response factors across various compounds. To monitor compound-independent response factors for F detection during this optimization, two compounds with vastly different chemical structures were chosen for the studies.

**Figure 2:**
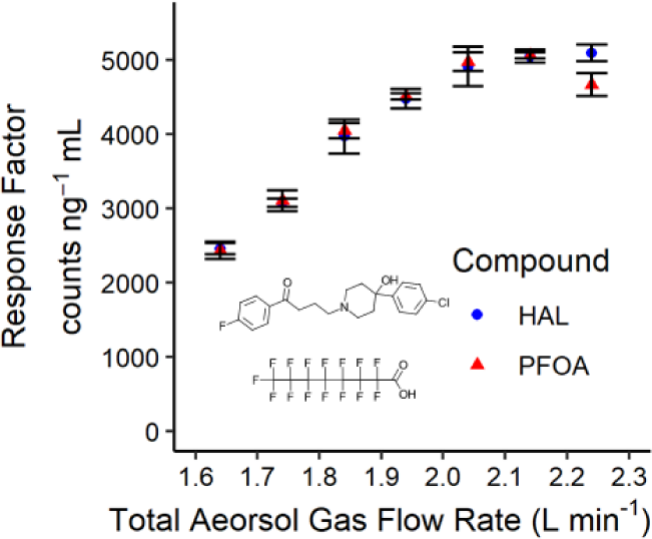
Effect of total aerosol gas flow rate on Sc(NO_3_)F(H_2_O)_4_^+^ → ScO_2_F^+^ response factors using ∼40 ng of F mL^–1^ from haloperidol and perfluorooctanoic acid and a single-pass spray chamber. Error bars show standard deviation from triplicate flow injections. Nebulizer Ar gas: 0.3 L min^–1^, plasma oxygen gas: 0.14 L min^–1^, Ar makeup gas: 1.2 to 1.8 L min^–1^, ICP power: 1300 W.

Figure 2 illustrates the impact of the total aerosol gas flow rate on the F response factors using a single-pass spray chamber. In these experiments, the plasma oxygen and nebulizer gas flow rates were constant while the makeup argon gas flow rate was varied. The response factors were measured via dividing the flow injection peak areas by F concentration in the injected sample. An increase in the response factor is evident up to a total aerosol gas flow rate of 2.04 L min^–1^ in Figure 2, while a plateau is reached at higher flow rates. The initial rise in signal intensity likely stems from enhanced aerosol transport efficiency to the plasma and improved generation and transmission of HF to the chemical ionization chamber, although the relative contributions of these factors remain unclear at this stage.

Notably, both compounds exhibit nearly identical response factors for F (with <2% difference) up to 2.14 L min^–1^, demonstrating quantitative decomposition of the compounds. However, a slight deviation from the compound-independent response factor is observed at 2.24 L min^–1^, marked by an 8.4% difference in F response factors between the two compounds. This suggests that aerosol gas flow rates exceeding 2.14 L min^–1^ may induce excessive plasma cooling, potentially compromising the compound breakdown efficiency. Based on Figure 2, the total aerosol gas flow rate of 2.04 L min^–1^ was selected to maximize sensitivity while maintaining species-independent response.

**Figure 3:**
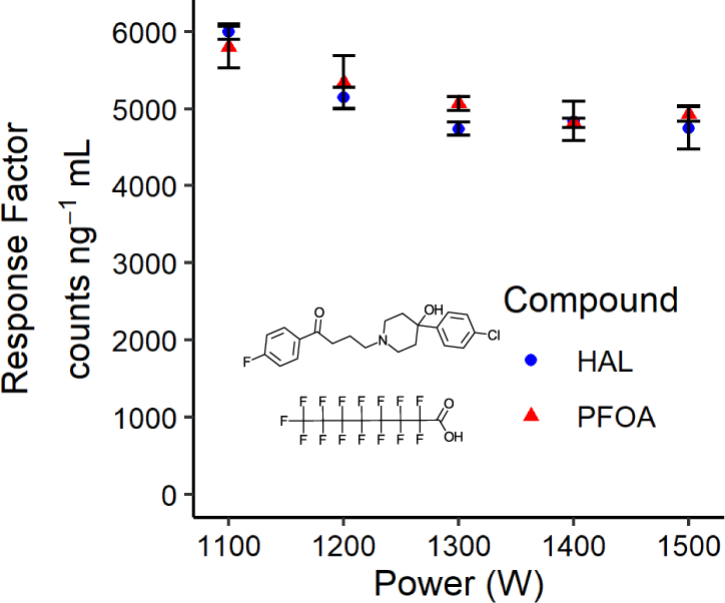
Effect of ICP RF power on Sc(NO_3_)F(H_2_O)_4_^+^ → ScO_2_F^+^ response factors using ∼40 ng of F mL^–1^ haloperidol and perfluorooctanoic acid and a single-pass spray chamber. Error bars show standard deviations from triplicate flow injections. Total aerosol gas flow rate: 2.04 L min^–1^.

Figure 3 illustrates the effect of ICP RF power on F response factors, indicating a minor (17%) decrease from 1100 to 1300 W and a negligible change at higher power levels up to 1500 W. Moreover, the two compounds produce response factors that are <6% different from one another and no clear trend in differences of response factors is observed as a function of RF power. Therefore, we infer that RF power has a minimal effect on breakdown of compounds. For subsequent measurements, we selected 1300 W. While a slight reduction in intensity is observed in Figure 3 at this power level, the higher power level affords better plasma stability with the introduction of organic solvents.

### 3.2 Comparison of Operating Parameters to Those in Conventional ICP-MS

The trends in Figures 2 and 3 stand in stark contrast to those observed in ICP-MS/MS for F detection, highlighting fundamental differences between post-ICP chemical ionization-MS and conventional ICP-MS. To combat the extremely inefficient F^+^ formation in ICP-MS, recent endeavors have utilized in-plasma BaF^+^ formation by leveraging cool plasma conditions and concurrent introduction of barium into the plasma.^26−28^ The optimum conditions are achieved at aerosol flow rate of ∼1.3 L min^–1^ (injector size = 1.5 mm) albeit with a narrow operating window. Specifically, BaF^+^ signal is reduced by a factor ∼2 upon 0.05 L min^–1^ deviation from optimum flow rate and becomes undetectable with deviations >0.1 L min^–1^.^27,28^ Similarly, a factor of 2 variation in BaF^+^ signal is reported for ICP-MS/MS when RF power is varied by 200 W from the optimum 1300 W.^27,28^ These observations indicate that a tight control of plasma temperature is required in ICP-MS/MS to maintain a delicate balance of atomization and in-plasma polyatomic ion (BaF^+^) formation.

In contrast, ScNO_3_F(H_2_O)_4_^+^ formation in post-ICP chemical ionization remains unaffected within 0.2 L min^–1^ at the plateau region of Figure 2 and RF power has a negligible effect on ion formation as noted in Figure 3. In this approach, plasma only acts as reactor to produce HF which is the thermodynamically favorable product of recombination reactions with its production showing less susceptibility to plasma temperature variation. The ensuing ionization of HF also has negligible dependence on plasma temperature because the ionization occurs in cool postplasma flow via ion-neutral reactions with Sc(NO_3_)_2_(H_2_O)_n_^+^. These attributes make post-ICP chemical ionization-MS a more desirable analytical approach in regard to stability and robustness to plasma temperature fluctuations.

### 3.3 Effect of Sample Introduction System on Compound-Independent Response

Quantitative fluorine detection irrespective of compounds’ chemical structures is generally attained in post-ICP chemical ionization-MS,^30,32^ owing to complete breakdown of compounds within the plasma. However, the efficiency of transport of the analyte from solution to the ICP may be influenced by the physicochemical properties of the analytes, potentially imparting analyte-dependent F detection efficiency. Volatility is one factor affecting the transport efficiency for small molecules. Considering the wide-ranging volatilities of perfluorinated compounds,^35^ we investigated the effect of this property on response factors using two different spray chambers.

**Figure 4:**
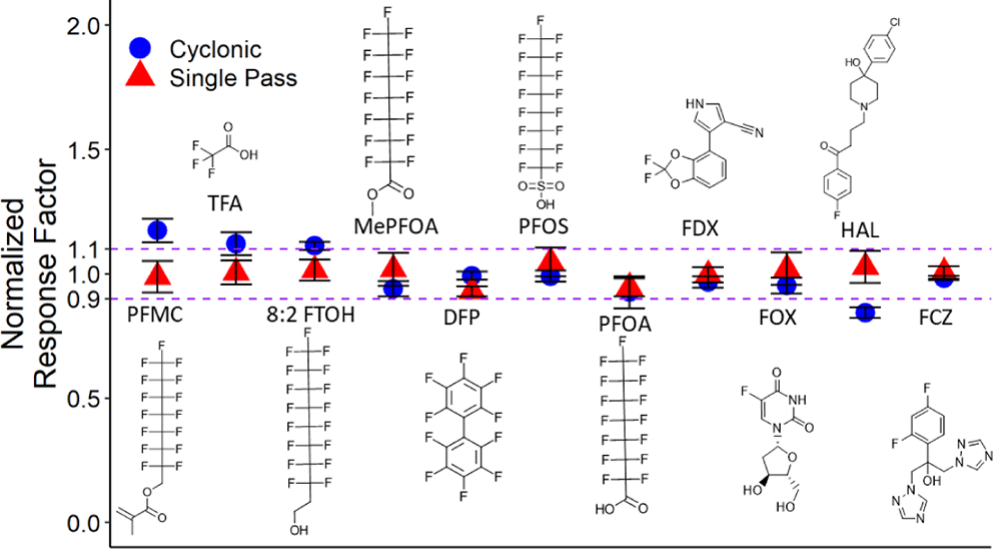
Effect of sample introduction on normalized F response factors from 11 compounds. Response factors are normalized to the average response factor for each spray chamber. Error bars reflect the standard deviation from triplicate flow injections.

Figure 4 contrasts the uniformity of the F response factor among various compounds (boiling points of 67–580 °C) using a cyclonic spray chamber and a single-pass spray chamber. The plasma operating parameters were constant in this comparison with a total aerosol gas flow rate of 2.04 L min^–1^ (including 0.14 L min^–1^ oxygen), while the ratio of the nebulizer and makeup argon gas was optimized for each spray chamber (Table S1). The response factors were determined by flow injections of compounds with F concentrations of 40–70 ng of F mL^–1^. The response factors were then normalized to the mean response factor for each spray chamber to facilitate comparisons.

The normalized response factors for the cyclonic spray chamber varied in the 0.84–1.17 range, while a narrower range of 0.93–1.05 was obtained using the single-pass spray chamber (Figure 4). Notably, the three compounds with highest normalized response factors using the cyclonic spray chamber were 1*H*,1*H*,2*H*,2*H*-perfluoro-1-decanol (8:2 FTOH), trifluoroacetic acid (TFA), and 1*H*,1*H*-perfluorooctyl methacrylate (PFMC) with boiling points <113 °C compared to the other 8 compounds with boiling points >150 °C. This suggests an enhanced transmission of volatile analytes to the plasma in cyclonic spray chamber relative to nonvolatile analytes, which is remedied using the single-pass spray chamber.

In a cyclonic spray chamber, the droplets follow a rotational path where large droplets deviate from the gas stream, collide with spray chamber walls, and are directed to drain. However, the volatile analytes can evaporate from the walls of the spray chamber, reentering the gas flow, though evaporation is not significant for nonvolatile compounds. Therefore, a larger response factor is observed for volatile analytes using a cyclonic spray chamber. In contrast, the single-pass spray chamber guides the spray directly toward the plasma, minimizing the collision of the droplets with the spray chamber walls. Therefore, the difference between the volatile and nonvolatile analytes is minimized.

The narrow range of normalized response factors (0.93–1.05) using the single-pass spray chamber in Figure 4 is also notable compared to that obtained by other total F quantitation methods. For example response factors of 110% for PFOS, 95% for PFOA, and 75% for 8:2FTOH were recently reported using CIC relative to ammonium fluoride, indicating a broader variability in F detection efficiency.^7^ Another study reported combustion efficiency of 73% for perfluorooctanesulfonic acid (PFOS) in CIC while a near complete (105%) combustion efficiency was measured for perfluorobutanoic acid (PFBA).^20^ To compare the response factors between PFBA and PFOS, we conducted a separate experiment using the single-pass spray chamber. The ratio of PFOS response factor to that of PFBA was 1.06 ± 0.08, indicating better species-independent response compared to CIC in agreement with the results of Figure 4. Notably, a recent optimization of GF-HR-MAS has shown normalized response factors of 0.7–1.3 for a broad range of PFAS compounds with potential to reduce it to 0.9–1.1 for narrower range of structures,^23^ further highlighting the advantages of the single-pass spray chamber post-ICP chemical ionization for quantitation of total EOF compared to other methods. Accordingly, a single-pass spray chamber was used for the remainder of the experiments.

### 3.4 Instrumental Analytical Figures of Merit

To characterize the instrumental analytical performance at optimized conditions, a calibration curve was constructed using PFOA as the test analyte in the 4–160 ng F mL^–1^ concentration range. An *r*^2^ = 0.9988 was obtained (Figure S2), indicating good linearity within close to 2 orders of magnitude variation in concentration. Instrumental LOD was measured using 7 injections of 9.8 ng of F mL^–1^ PFOA solution. An LOD of 2.2 ng F mL^–1^ was obtained based on 3 × SD/slope where SD was the standard deviation of the flow injection peak areas for the 7 injections and slope was taken from Figure S2. LOD was also calculated using flow injection peak heights. For this purpose, standard deviation of the baseline for 1 min was considered along with the slope of the calibration curve based on flow injection peak heights, providing an LOD of 2.1 ng mL^–1^ similar to the above value. This instrumental LOD is comparable to 3 ng F mL^–1^ reported for CIC,^19^ while absolute F LOD of 0.066 ng F is about 10-fold better than that of CIC as a result of much smaller injection volume. The LOD for post-ICP chemical ionization is also 10-fold better than those reported using ICP-MS/MS (22–60 ng F mL^–1^).^26−28^ The triplicate flow injections in experiments of Figure 4 show 2.2–6.6% RSD, indicating good repeatability. Noting the favorable analytical performance metrics, we proceeded to develop a total EOF quantitation method on food contact paper products via methanolic extraction followed by SPE as described below.

### 3.5 Characterization of PFAS Extraction by SPE

To investigate quantitative extraction of PFAS by SPE, we first characterized the recovery of analytes from a pure solvent. Five compounds (see Table 1) were spiked individually into 40 mL of methanol producing concentrations ∼4.4 ng F mL^–1^ and were subjected to SPE as described in the Materials and Methods section. Table 1 also shows the quantitation results using a calibration curve based on perfluorooctanoic acid and consideration of an 8-fold preconcentration after SPE. Generally, recoveries of ∼100% are observed. However, 1*H*,1*H*-perfluorooctyl methacrylate (8:2 FTOH) has a reduced recovery of 48%, attributed to volatility of this analyte and loss during the SPE drying process. It is notable that trifluoroacetic acid and 1*H*,1*H*-perfluorooctyl methacrylate are also volatile analytes. However, low p*K*_a_ of trifluoroacetic acid ensures ionization in solution and its capture by the anion exchange resin while 1H,1H-perfluorooctyl methacrylate likely is retained by the polymeric reversed phase SPE. The other 2 compounds have an ionic form and interact better with the Strata-X-AW bed.

**Table 1:** Recovery of Compounds Spiked into 40 mL of Methanol After SPE Measured Using a Calibration Curve Based on PFOA^a^

Compound	Spiked F concentration in 40 mL methanol (ng mL^–1^)	Measured F concentration after SPE (ng mL^–1^)	Preconc. Factor by SPE	Calculated F concentration in 40 mL methanol (ng F mL^–1^)	Recovery (%)
Perfluorobutanoic acid	4.3	34.9 ± 1.7	8	4.4 ± 0.2	101.4 ± 4.9
1*H*,1*H*-perfluorooctyl methacrylate	4.7	37.5 ± 1.2	8	4.7 ± 0.1	99.8 ± 3.1
1*H*,1*H*,2*H*,2*H*-perfluoro-1-decanol	4.1	15.9 ± 0.4	8	2.0 ± 0.0	48.3 ± 1.1
Perfluorooctanoic acid	3.8	30.4 ± 0.1	8	3.8 ± 0.0	100.0 ± 0.3
Trifluoroacetic acid	4.7	38.3 ± 2.4	8	4.8 ± 0.3	101.6 ± 6.5

a The standard deviations are from triplicate injections of the final preconcentrated sample.

To examine the SPE extraction reproducibility and potential matrix effects from easily ionizable elements, five 40 mL methanolic extractions of a food contact paper product (paper plate) were pooled, producing 200 mL of methanolic extraction solution. The pooled solution was shaken to homogenize and was subsequently split into five 40 mL aliquots. Three aliquots were used for the SPE reproducibility characterization. The aliquots were subjected to SPE process and F concentrations in the final preconcentrated samples were measured using a calibration curve based on PFOA. As indicated in Table 2, the three aliquots of the paper extract yielded an average of 5.3 ng mL^–1^ F with a reproducibility of 1.6% RSD, indicating good reproducibility of the SPE and measurement processes.

**Table 2:** Total EOF Measurements Using Paper Plate Methanolic Extracts as Matrix and Calibration Curve Based on PFOA^a^

Sample	Spiked F concentration in 40 mL methanolic extract (ng mL^–1^)	Measured F concentration after SPE (ng mL^–1^)	Preconc. factor by SPE	Calculated F concentration in 40 mL methanolic extract (ng mL^–1^)
Paper plate methanolic extract aliquot 1	0	42.1 ± 1.2	8	5.3 ± 0.2
Paper plate methanolic extract aliquot 2	0	43.3 ± 3.4	8	5.4 ± 0.4
Paper plate methanolic extract aliquot 3	0	42.1 ± 2.0	8	5.3 ± 0.2
Paper plate methanolic extract aliquot 4 spiked with 100 mM sodium acetate	0	42.8 ± 1.3	8	5.4 ± 0.2
40 mL methanol spiked with 100 mM sodium acetate	0	N.D.^b^	8	N.D.^b^
Paper plate methanolic extract aliquot 5 spiked with PFOA	7.2	98.2 ± 3.4	8	12.3 ± 0.4

a Standard deviations are from triplicate injections.

b Not detected.

A potential matrix effect in F detection using post-ICP-chemical ionization-MS may arise from easily ionizable elements, such as sodium.^27^ To determine the effectiveness of SPE in removing such interference, the fourth aliquot of the pooled methanolic extract was spiked with 100 mM sodium acetate prior to SPE. A 100 mM sodium acetate in pure methanol was also created as a blank and subjected to SPE to ensure that the sodium acetate does not include fluorinated contaminants. As noted in Table 2, F was not detected in the blank and the concentration of F in the sodium spiked sample was not statistically different than that of the unspiked sample, confirming that easily ionizable elements do not affect the measured total organic F in the sample after SPE extraction.

Finally, an analyte spike recovery experiment was conducted. The fifth aliquot of the pooled extract was spiked with PFOA at a concentration of 7.2 ng F mL^–1^. The aliquot was subjected to SPE and analyzed, yielding a total F concentration of 12.3 ng F mL^–1^ as noted in Table 2. Subtracting the original 5.3 ng F mL^–1^ prior to spike results in a recovery of 97.2 ± 7.8%, indicating good analyte recoveries from paper extract matrix.

Based on the instrumental LOD noted above and the 8-fold preconcentration by SPE, the method LOD is estimated to be 0.26 ng F mL^–1^ in the methanolic extract. Given the 9 cm^2^ samples (assuming coating on one side only) and 40 mL of extraction volume, an LOD of 1.2 ng F cm^–2^ is obtained for paper products. This value is 2 orders of magnitude lower than 100 ng F cm^–2^ specified by Danish Ministry of the Environment and Food for product labeling,^11^ indicating applicability to regulatory matters. This value also compares favorably to those of other methods. For example, Schultes et al. reported a method for EOF quantitation in food contact paper products using CIC with an LOD of 40–70 ng F cm^–2^.^9^ LODs of ∼300 ng F cm^–2^ have also been reported for F quantitation of food packaging products using PIGE.^13,36^

### 3.6 Total Extractable Organic F on Food Contact Paper Products

With promising analytical performance noted above, the method was applied to quantitation of EOF in nine food contact products, resulting in 22.0–362.7 ng F cm^–2^ (assuming coating on only one side) as shown in Table 3. Notably, the majority of the samples have EOF < 100 ng F cm^–2^. In comparison, total EOF of 220–490 ng F cm^–2^ has been reported in food contact papers,^9^ while 5 out of 9 samples in this study fell below the method LOD of 40–70 ng F cm^–2^, hindering quantitation. Another study reported 60–2340 ng F g^–1^ for 7 food contact paper products with majority of samples in 160–500 ng F g^–1^ range.^7^Table 3 also provides our results in weight units using the mass of the samples, showing slightly higher levels but in general range of total EOF by other methods. It is of note that the method LOD in our studies translates to 40 ng F g^–1^ using an average weight of 0.27 g for samples, indicating the capability of this method to quantify the EOF for a wide range of samples. Clearly, larger sample sizes or further preconcentration would improve the method LOD to measure even lower concentrations. For example, one study noted above^7^ employed 4 paper disks of 3.17 cm i.d. (3.5-fold more sample surface than our method) and a final solution of volume of 1 mL (additional 5 fold precontraction relative to our method).

**Table 3:** Total Extractable Organic Fluorine in Food Contact Papers^a^

Food Contact Paper Product	Measured F in SPE extract (ng F mL^–1^)	Precon. Factor by SPE	Calculated F in 40 mL Extract(ng F mL^–1^)	F on Sample (ng F cm^–2^)	Average Weight of Paper Product (g)	F on Sample (ng F g^–1^)^b^
Burger Box	90.2 ± 2.7	8	11.3 ± 0.3	50.1 ± 1.50	0.3820 ± 0.0022	1180 ± 36
Coffee Cup A	54.4 ± 1.7	8	6.8 ± 0.2	30.2 ± 0.9	0.2900 ± 0.0025	937 ± 30
Coffee Cup B	71.7 ± 2.0	8	9.0 ± 0.3	39.8 ± 1.1	0.2681 ± 0.0047	1336 ± 45
Fries Box	90.8 ± 1.4	8	11.4 ± 0.2	50.5 ± 0.8	0.3586 ± 0.0023	1267 ± 21
Paper Plate	39.6 ± 2.3	8	5.0 ± 0.3	22.0 ± 1.3	0.2618 ± 0.0056	756 ± 47
Popcorn bag A	65.3 ± 0.4	0.8^c^	81.6 ± 0.6	362.7 ± 2.6	0.1405 ± 0.0054	23233 ± 912
Popcorn bag B	54.9 ±0.9	2.67^c^	20.6 ±0.3	91.4 ±1.4	0.1375 ±0.0034	5982 ±175
Straw	43.8 ±2.2	8	5.5 ±0.3	24.4 ±1.2	0.2810 ±0.0045	780 ±41
To Go Box	46.9 ±2.0	8	5.9 ±0.2	26.0 ±1.1	0.3488 ±0.0050	672 ±30

a Standard deviations are based on triplicate flow injections.

b Calculated based on average weight of 3 cm × 3 cm sample for each product.

c The SPE of popcorn bag samples resulted in concentrations beyond the upper end of the calibration curve. Therefore, these samples were diluted prior to analysis. The preconcentration factors reflect this dilution.

## 4 Conclusions

A plasma-based MS method for quantifying total extractable organic F is presented in this work, with an application to food contact paper products. The method utilizes post-ICP chemical ionization-MS with Sc(NO_3_)_2_(H_2_O)_*n*_^+^ reagent ions to ionize HF formed in postplasma flow upon injection of fluorinated compounds. Investigation of plasma operating parameters shows that ion generation has far reduced dependence on critical factors, such as aerosol gas flow rate and plasma power compared to that observed in ICP-MS/MS, making the new method more resilient to plasma temperature fluctuations and affording improved stability. The high aerosol gas flow rate compared with that in ICP-MS/MS also helps improve analyte transport efficiency to the plasma. Notably, the use of a single-pass spray chamber mitigates volatility biases in the detection technique, improving the accuracy of F quantitation from a variety of compounds. Rapid flow injections provide detection limits in low ng F mL^–1^, competitive with those of widely used CIC and 10-fold better than that of ICP-MS/MS. Coupled with SPE, this detection limit can be improved via preconcentration to be applicable to emerging needs. In this work, we demonstrated application to food contact paper products. The experiments yielded total EOF values in the range of those reported by other methods in the literature. These results complement quantitation of fluorinated compounds without analyte-specific standards using LC reported in our previous work,^32^ showcasing the post-ICP chemical ionization as a versatile approach for facile organofluorine quantitation in both total F and species-specific quantitation modes.

## Supporting Information Available

Table of instrumental parameters, pictures of spray chambers, and calibration curve for F detection (PDF)
