# Supplementary material for: Post-ICP Chemical Ionization MS for Total Extractable Organic Fluorine Quantitation
Source: ACS Omega. 2024 Nov 5;9(46):46634–42. doi: 10.1021/acsomega.4c09483 (PMC11579928; doi:10.1021/acsomega.4c09483)
Supplement: Supplementary file 1 — ao4c09483_si_001.pdf [file ao4c09483_si_001.pdf]

Supporting Information for

Post-ICP Chemical Ionization MS for Total Extractable Organic Fluorine Quantitation

Samuel White and Kaveh Jorabchi*

Department of Chemistry, Georgetown University, Washington, DC 20057

*Corresponding author: kj256@georegtown.edu

Table of Contents:

Page 2: Table S1 – Instrumental parameters

Page 3: Figure S1 – Picture of cyclonic spray chamber and single-pass spray chamber

Page 3: Figure S2 – Calibration curve for F quantitation

Table S1. Instrument parameters

Solvent pump	Agilent 1200 LC pump
Solvent flow rate	50 µL min^{-1}
Mobile phase solvent	80:15:5 ACN:W:Concentrated ammonium hydroxide
Injection volume	30 µL
Cyclonic spray chamber	
Nebulizer	Meinhard HEN-90-A0.1
Spray chamber	Meinhard cyclonic spray chamber
Nebulizer argon gas	1.4 L min^{-1}
Aerosol makeup gas	0.5 L min^{-1} Ar + 0.14 L min^{-1} O_2
Single pass spray chamber	
Nebulizer	Glass Expansion MicroMist HE U
Spray chamber	Glass Expansion single-cell spray chamber
Nebulizer argon gas	0.3 L min^{-1}
Aerosol makeup gas	1.2-1.8 L min^{-1} Ar + 0.14 L min^{-1} O_2
ICP	
ICP generator	PerkinElmer NexIon
RF power	1100-1500 W
Plasma argon gas	14 L min^{-1}
Auxiliary argon gas	1.2 L min^{-1}
ICP torch injector size	2 mm
Plasma sampling cone	4 mm Nickel
Chemical Ionization Chamber	
Gas evacuation rate	4 L min^{-1}
Nitrogen input flow	2 L min^{-1}
Oxygen input flow	0.56 L min^{-1}
Quartz recombination tube	57 mm × 1/4 ″ o.d. × 4 mm i.d.
Plasma ion deflectors	400 V top electrode, ground bottom electrode
Nanospray tip diameter	~5 µm
Nanospray electrolyte	1 mM scandium nitrate in water
Spray potential	1400 V
Ion sampling steel tube	27 mm × 1/8″ o.d. and 2 mm i.d
Mass spectrometer	Sciex API 3000 triple quadrupole
Multiple reaction monitoring	
MS/MS transitions (dwell times)	$ScNO_3F(H_20)_4^+$ m/z 195 →ScO_2F^+ m/z 96 (500 ms)<br>$Sc(NO_3)_2(H_20)_3^+$ m/z 223 → $ScO_2NO_3^+$ m/z 139 (100 ms)
Declustering potential	10 V
Focusing potential	100 V
Entrance potential	10 V
Focusing lens 2	-12 V
Collision energy	40 eV
Collision cell exit potential	5 V
Collision gas setting	12
Q1 resolution	Unit
Q3 resolution	Low

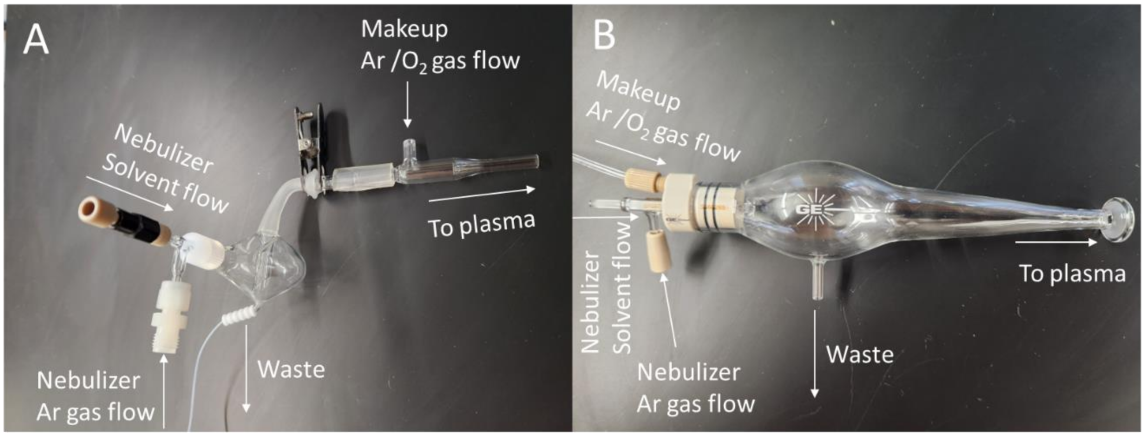

Fig. S1. A) cyclonic spray chamber and B) single-pass spray chamber

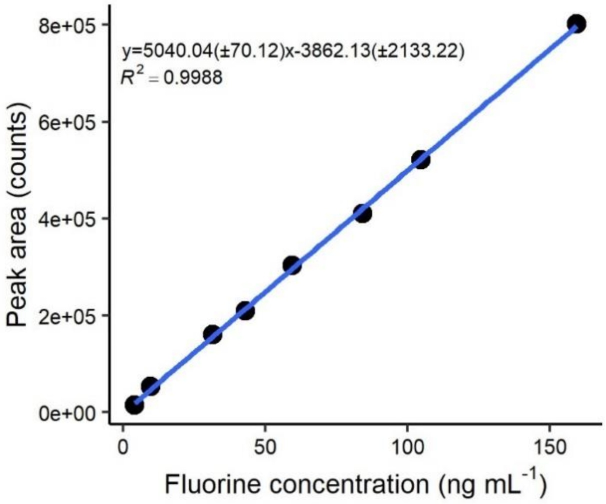

Fig. S2. Calibration curve using PFOA ranging from 4 to 160 ng F mL^{-1}. A weight of 1/y was applied for linear regression. The numbers in parentheses within the regression formula show the standard errors of slope and intercept.
